# Supplementary material for: Characterization of international partnerships in global retinoblastoma care and research: A network analysis
Source: PLOS Glob Public Health. 2021 Dec 16;1(12):e0000125. doi: 10.1371/journal.pgph.0000125 (PMC10021644; doi:10.1371/journal.pgph.0000125)
Supplement: S2 File — The questionnaire used in this study. (DOCX) [file pgph.0000125.s002.docx]

# SUPPLEMENTAL INFORMATION

## Supplemental File S2. Network Analysis Survey

Network Analysis Survey

Purpose

The purpose of this survey is to find out information about how retinoblastoma treatment

centers relate to, work with, and coordinate among each other.

Confidentiality

All of the information you provide will be kept confidential. Your responses will be

combined with the responses of other partners.

Q1 Name:___________________________________

Q2 For each of the activities listed below, indicate which of the treatment

centers you have engaged with in the last 12 months.

| Activities | Partner Retinoblastoma Treatment Center(s) | In what year did this collaboration begin? |
| --- | --- | --- |
| Type of Interactions  • patient referrals, sent  • patient referrals, received  • research, initiated by your center  • research, initiated by other center  • patient consultations, initiated by your center  • patient consultations, initiated by other center  • twinning/capacity building projects, initiated by your center  • twinning/capacity building projects, initiated by your center |  |  |
| Sharing Information  • Information received  • Information shared |  |  |
| Sharing Tangible Resources (e.g.funds, equipment, materials, etc.)  • Resources received  • Resources shared |  |  |
| Joint Planning |  |  |
| Formal Agreement / Memorandum of  Understanding |  |  |
| Other type of Activity (Specify): |  |  |

Q3 For each of the activities listed below, please indicate any other organizations (e.g. NGOs, patient advocacy groups) you have engaged within the last 12 months.

| Activities | Other partner organizations | In what year did this collaboration begin? |
| --- | --- | --- |
| Type of Interactions  • patient referrals, sent  • patient referrals, received  • research, initiated by your center  • research, initiated by other center  • twinning/capacity building projects, initiated by your center  • twinning/capacity building projects, initiated by your  center |  |  |
| Sharing Information  • Information received  • Information sent |  |  |
| Sharing Tangible Resources (e.g. funds, equipment, materials, etc.)  • Resources received  • Resources sent |  |  |
| Joint Planning |  |  |
| Formal Agreement / Memorandum of  Understanding |  |  |
| Other type of Activity (Specify): |  |  |

Q4 Please indicate how often the reported activities took place over the last 12 months.

| Organization & Activity | Once or twice | Every few months | Monthly | Weekly | Daily | Don’t  know |
| --- | --- | --- | --- | --- | --- | --- |
| [pre-populated based on responses to Q2 & Q3] |  |  |  |  |  |  |
| e.g. “patient referrals, sent – hospital name” |  |  |  |  |  |  |

Q5 What type of information was shared (i.e., funding opportunities, policy changes, treatment protocols, etc.)?

| Organization & Information | Type of Information |
| --- | --- |
| [pre-populated based on responses to Q2 & Q3] |  |
| e.g. “information received – hospital name” |  |

Q6 What type of tangible resources were shared? Was there a Formal Agreement or Memorandum of Understanding (MoU) governing the sharing of resources?

| Organization & Information | Type of Resource(s) | Formal Agreement/MoU |
| --- | --- | --- |
| [pre-populated based on responses to Q2 & Q3] |  | Yes  No  Don’t know  Not applicable |
| e.g. “resources  received – hospital name” |  | Yes  No  Don’t know  Not applicable |

Q7 Please indicate the purpose of the joint planning that took place (e.g to plan training, meetings, communication)?

| Organization & Information | Joint Planning |
| --- | --- |
| [pre-populated based on responses to Q2 & Q3] |  |

Thank you for completing the social network survey.
