# Supplementary material for: Characterization of international partnerships in global retinoblastoma care and research: A network analysis
Source: PLOS Glob Public Health. 2021 Dec 16;1(12):e0000125. doi: 10.1371/journal.pgph.0000125 (PMC10021644; doi:10.1371/journal.pgph.0000125)
Supplement: S3 File — The semi-structured interview guide used in this study. (DOCX) [file pgph.0000125.s003.docx]

## Supplemental File S3. Semi-structured Interview Guide

**Questions aimed at general history/description of partnerships**

Q1a Please describe your treatment center’s history with national and/or international partnerships with respect to retinoblastoma.

Q1b Why participate in such partnerships? Can you comment on motivations?

Q2a What is/are your strongest partnership(s)? How did this/these connection(s) come to be?

Q2b Can you comment on the duration/intensity/intimacy/reciprocity of your interactions with your strongest partners?

Q2c Can you elaborate on your activities with your strongest partners?

Q3a What is/are your weakest partnership(s)? How did this/these connection(s) come to be?

Q3b Can you comment on the duration/intensity/intimacy/reciprocity of your interactions with your weakest partners?

Q3c Can you elaborate on your activities with your weakest partners?

**Questions aimed at elucidating details of specific partnerships**

Q4 How would you describe the strength of the partnership between your treatment center and [treatment center of interest].

Q5 How would you describe the level of engagement of each partner? Would you describe this as a close partnership? Does any institution lead the partnership?

Q6 Can you comment on the financial resources necessary (if any) for participation in partnership activities?

Q7 Can you elaborate on any formal or informal agreements that govern your interactions with [treatment center of interest]? In what context are they useful?

**Questions aimed and understanding benefits and challenges of partnerships**

Q8 What do you believe has been the greatest impact of your partnership with [treatment center of interest]. Can you give any examples related to healthcare capacity/patient survival/knowledge exchange/resources/planning/other?

Q9a What are some benefits of strong partnerships? Challenges?

Q9b What are some benefits of weak partnerships? Challenges?

Q10a Describe two examples of challenges faced in partnerships between your treatment center and other treatment centers in general.

Q10b Has your treatment center addressed any of the challenges? What steps were taken to mitigate them?

Q10c If no challenges were addressed, how might your treatment center address them in the future?
