# Supplementary material for: Characterization of international partnerships in global retinoblastoma care and research: A network analysis
Source: PLOS Glob Public Health. 2021 Dec 16;1(12):e0000125. doi: 10.1371/journal.pgph.0000125 (PMC10021644; doi:10.1371/journal.pgph.0000125)
Supplement: S4 File — Summary data of egos represented in this study, presented by geographical region and country. (DOCX) [file pgph.0000125.s004.docx]

## Supplemental File S4. Egos by Region and Country

| **Geographical Region** | **Country** | **Egos** | | | |
| --- | --- | --- | --- | --- | --- |
|  |  | **n** | | **%** | |
| East Asia and Pacific |  | 21 |  | 38% |  |
|  | *China* |  | *18* |  | *32%* |
|  | *Australia* |  | *2* |  | *4%* |
|  | *Hong Kong* |  | *1* |  | *2%* |
| Europe and Central Asia |  | 8 |  | 14% |  |
|  | *Czech Republic* |  | 1 |  | *2%* |
|  | *Denmark* |  | 1 |  | *2%* |
|  | *Estonia* |  | 1 |  | *2%* |
|  | *France* |  | 1 |  | *2%* |
|  | *Hungary* |  | 1 |  | *2%* |
|  | *Spain* |  | 1 |  | *2%* |
|  | *Switzerland* |  | 1 |  | *2%* |
|  | *Ukraine* |  | 1 |  | *2%* |
| Latin America and Carribean |  | 3 |  | 5% |  |
|  | *Brazil* |  | 1 |  | *2%* |
|  | *Honduras* |  | 1 |  | *2%* |
|  | *Mexico* |  | 1 |  | *2%* |
| Middle East and North Africa |  | 3 |  | 5% |  |
|  | *Iran* |  | 1 |  | *2%* |
|  | *Israel* |  | 1 |  | *2%* |
|  | *Jordan* |  | 1 |  | *2%* |
| North America |  | 8 |  | 14% |  |
|  | *Canada* |  | 5 |  | *9%* |
|  | *United States of America* |  | 3 |  | *5%* |
| South Asia |  | 5 |  | 9% |  |
|  | *India* |  | 5 |  | *9%* |
| Sub-Saharan Africa |  | 8 |  | 14% |  |
|  | *Kenya* |  | 2 |  | *4%* |
|  | *Burundi* |  | 1 |  | *2%* |
|  | *Cote D'ivoire* |  | 1 |  | *2%* |
|  | *Gambia* |  | 1 |  | *2%* |
|  | *Ghana* |  | 1 |  | *2%* |
|  | *Nigeria* |  | 1 |  | *2%* |
|  | *Zimbabwe* |  | 1 |  | *2%* |
| **TOTAL** | | 56 | | 100% | |
