# Supplementary material for: Characterization of international partnerships in global retinoblastoma care and research: A network analysis
Source: PLOS Glob Public Health. 2021 Dec 16;1(12):e0000125. doi: 10.1371/journal.pgph.0000125 (PMC10021644; doi:10.1371/journal.pgph.0000125)
Supplement: S5 File — Summary network density statistics by overall and individual (in-degree and out-degree, where relevant) interactions. (DOCX) [file pgph.0000125.s005.docx]

## Supplemental File S5. Network Density

| **Entire Network** | **Density** |
| --- | --- |
| All types of Interactions | 1.30% |
| Referalls Sent | 0.52% |
| Referalls Received | 0.50% |
| Information Shared | 0.24% |
| Information Received | 0.16% |
| Consultations (out-degree) | 0.22% |
| Consultations (in-degree) | 0.25% |
| Research (out-degree) | 0.21% |
| Research (in-degree) | 0.19% |
| Resources Shared | 0.13% |
| Resources Received | 0.14% |
| Twinning (out-degree) | 0.08% |
| Twinning (in-degree) | 0.09% |
| Joint planning | 0.15% |
| Other | 0.26% |
