# Supplementary material for: Characterization of international partnerships in global retinoblastoma care and research: A network analysis
Source: PLOS Glob Public Health. 2021 Dec 16;1(12):e0000125. doi: 10.1371/journal.pgph.0000125 (PMC10021644; doi:10.1371/journal.pgph.0000125)
Supplement: S6 File — Network diagrams depicting joint planning and other activities. (DOCX) [file pgph.0000125.s006.docx]

## Supplemental File S6. Network diagrams for joint planning and other activities.

Network diagrams depicting (a) joint planning and (b) other activities.
