# Supplementary material for: Characterization of international partnerships in global retinoblastoma care and research: A network analysis
Source: PLOS Glob Public Health. 2021 Dec 16;1(12):e0000125. doi: 10.1371/journal.pgph.0000125 (PMC10021644; doi:10.1371/journal.pgph.0000125)
Supplement: S7 File — Resulting themes and supporting participant quotes from the qualitative analysis. (DOCX) [file pgph.0000125.s007.docx]

## Supplemental File S7. Themes and Sample Quotes.

| **Themes** | **Subtheme** | **Sample Quotes** |
| --- | --- | --- |
| **Conceptualization of partnership** | Informal partnership | Partnership is too vague of a term, nothing is formal. |
|  |  | Up until now it has been a very informal network or relationship with the other treatment centers. |
|  | Formal partnership | If we’ve got a more formalized alliance, we would be able to make a more measured and unified response. |
|  |  | In the last three months, we have made extraordinary advances in formalizing this network and the meeting will be not clinical but strategic, so talking about how do we deliver all the suite of treatments for retinoblastoma. |
|  | Strong ties | Very close [in terms of engagement], because it’s based on the personal contacts mostly. We meet in the meetings and find that we have common interest and as a person we are very good friends, that is probably why we have such good cooperation. |
|  |  | I think the stronger the partnership, the higher the investment of your time and energy would be. |
|  | Weak ties | We learn from other centers, what they are doing, the new techniques, that we learn, not necessarily by partnerships but even during scientific meetings, ocular oncology meetings, retinoblastoma meetings. We learn what others are doing and as you may know, there is a big evolvement, big change in the way we treat retinoblastoma. Not real partnerships, being in contact with each other, listening to each other, learning from each other. |
|  |  | A lot of times you just need a quick answer to something, that doesn’t require much of the other partner, you just need access to expertise to answer a question or something, without any kind of obligation for ongoing, extensive investment on either side. |
|  | Equal leadership | It is very important that if the partnership is going to work, it needs to be a very equal partnership; I don’t think anyone wants any particular group to be the leader. |
|  | Unequal leadership | [The partner] is a bigger center, a more experienced center, is doing much more research on retinoblastoma...So, I would consider [clinician name and team] as the leading team, they are a leading team in the world also. |
|  |  | We’re leading the partnership, meaning we’re reaching out to them and they’re responding to that. They’re not the ones pushing it forward, we’re the ones doing that. |
| **Primary motivations to collaboration** | | I think it is very important to work thinking about the patient for the first goal, that is something that we should all keep in mind. |
|  |  | I guess because we are passionate about children with retinoblastoma and treatments not so straightforward anymore. And to be able to give all children the opportunity to access all the treatments that are available. |
|  |  | I think the greatest impact, because the, if you come from a small country, we would never have enough experience to give my patients the perfect treatment, in the difficult cases. I have a good friend, good expert, that I can ask for advice. |
| **Common challenges to collaboration** | | I think ego can get in the way, which is really unfortunate because at the end of the day we’ve all got the same goal and that is the children. And serving them, and treating them for the best possible outcome. |
|  |  | I would just say communication between two places in rural Africa is challenging |
|  |  | But I think that’s a big challenge. Maybe also a challenge, where other groups don’t have the same access to resources. |
|  |  | [The partnership is] always in danger because of political situation. |
|  |  | I mean strong partnerships need lots of communication and that requires a lot of time and investment. |
| **Benefits of partnership** | **High-income country perspective** | For example, with our education program we hold in [low-and-middle income country] we changed the survival of the patients there. Or, with [low-and-middle income country], we also improve the health of all the patients they send to us. Same in [low-and-middle income country]. Regarding [high income country], we usually are more facing investigational, papers, reports, things like that. |
|  | **Low-and-middle-income country perspective** | The other area of partnerships and collaborations [benefit] might be genetic studies. |
|  |  | [International NGO] were in our city… I began, with a number of international volunteers, working in different specialties of ophthalmology…the following year I went to [low-and-middle income country] to have some training … then had the opportunity, on the International Council of Ophthalmology sponsored fellowship to [low-and-middle income country]…[these experiences] got me into relationships with people...so, that if I had a case I had issues with, I discussed with [them]…Then, we pushed on…Our partners are beyond the hospital, the pharmaceutical companies, mass media people, philanthropies who need to help us with drugs, payments and all sort of things. Now this is outside of our hospital, so I began to talk to these groups, at different opportunities, because they were, when they invite me to talk to them about blindness, and anything, I will find a way to talk about retinoblastoma and get them to help. |
|  |  | You can also ask for second opinions from people who are experts in some fields of retinoblastoma I don’t know. Research is very important. I think one of the most important things in a training hospital is to do research in order to motivate our trainees, our fellows, to ask questions and make advances in medicine. |
