# Supplementary material for: Characterization of international partnerships in global retinoblastoma care and research: A network analysis
Source: PLOS Glob Public Health. 2021 Dec 16;1(12):e0000125. doi: 10.1371/journal.pgph.0000125 (PMC10021644; doi:10.1371/journal.pgph.0000125)
Supplement: Attachment — Submitted filename: Dimaras Response to Reviewers PLOS GPH v1.pdf [file pgph.0000125.s009.pdf]

SickKids® Ophthalmology & Vision Sciences UNIVERSITY OF TORONTO

Catherine Kyobutungi, MD, PhD
Editor-in-Chief, PLOS Global Public Health
African Population and Health Research Center
Nairobi, Kenya

15 October 2021

Dear Dr. Catherine Kyobutungi,

RE: "Characterization of international partnerships in global retinoblastoma care and research: a network analysis" by Girdler et al.

Thank you for the opportunity to submit a revision of our manuscript entitled "Characterization of international partnerships in global retinoblastoma care and research: a network analysis". We respond to the reviewer comments in a point-by-point fashion below.

Reviewer 1:

Thanks for the opportunity to review such an interesting piece of work. The authors did an excellent job in collecting valuable data on a relevant global health topic, which is eye care (or more specifically retinoblastoma care). The introduction, the qualitative analysis and the discussion are very stimulating.

Response: Thank you for these positive comments.

The network analysis is also potentially very rich and interesting. However, I believe the authors fail to make a sufficiently good use of the data available in terms of network statistics. Additionally, the integration between the qualitative and the quantitative components of the study could be made more explicit in the discussion. I believe that conducting and reporting a few additional standard network analysis procedure would offer additional insights that can then feed into the discussion, adding interesting content and linking nicely the qual and quant components of this mixed methods paper.

Response: We have improved upon these areas by following your specific recommendations below.

Below a few specific comments on different paper sections:
Methodology
- Add network questionnaire in Appendix

Response: We now include the network questionnaire as new Supplemental File S2.

- For the network analysis methods, an accessible "guide" is provided in Blanchet & James (2012), https://doi.org/10.1093/heapol/czr055. It is worth having a look (and potentially referencing) if you didn't see it earlier.

Response: Thank you for recommending this reference. We cited this guide as follows: "The study methodology was aligned with the 3 main stages of social network analysis for health services research: (i) define actors and members in the network; (ii) define relationships between actors, and (iii) analyse the structure of the system" (Page 7, lines 112-114). The methods section was also re-organized to highlight the 3 stages of analysis (Pages 8-11, lines 129-236).

- How did you choose which organisations (egos) to invite for the interview, of those who replied to the quant survey?

Response: We have added these methodological details as follows: "Key informants were chosen using the maximum variation method; to do this we reviewed responses to the network questionnaire and invited respondents who represented different geographical regions and had diverse experiences with number and types of activities (as described above) or partners (i.e. hospitals, other organizations, local or international, etc.)." (Page 10, lines 188-192).

Results

- The results offer a valid description of the data. However, as it is, the paper fails to exploit the rich and interesting data that and most of all fails to integrate properly the "network analysis" with the qualitative analysis (see reference above, Blanchet & James 2012). Two low hanging fruits directly from the network analysis toolbox that could provide interesting insights for the discussion are:

1. Explore a few centrality measures, discussing the characteristic of the most central organizations in the networks and sub-networks identified by the study: who are these powerful organisations? What is their role? How does this affect the overall functioning of the network?

Response: We now report on the measures of centrality as follows: "Measures of centrality generally indicated that larger treatment centers in HICs were most central to networks of overall collaboration, sharing of information and tangible resources, joint planning, and patient referrals. Three centers, in particular, were most central to the network of overall partnership, one in North America, one in Europe, and one in East Asia, each representing large tertiary centers with well-established expertise in retinoblastoma. Interestingly, the center in East Asia had nearly equal measure of in- and out-degree centrality (17 versus 16), whereas the European and North American centers had much larger out-degree centrality relative to much smaller in-degree centrality (13 versus 5 and 30 versus 5, respectively)." (Page 14, lines 279-287).

2. Potentially, it could be interesting explore the relationship between separate collaborative activities (which I assume are asked non-exclusively in the questionnaire). This could be explored graphically or – in a more straightforward way - more analytically, in terms of association between different networks of collaborative activities. This latter point could be addressed with simple adjacency matrix correlations, or using a more involved inferential approach (e.g. Exponential Random Graph Models). Questions that could be addressed with such tools are, for example: "How well does having information sharing predict having a patient referral network?", or "Does sharing resources predict having patient shared consultation?"

Response: We would like to thank the reviewer for this suggestion. We agree this would, indeed, be an interesting exploration of our data. However, given the scope of the current paper, we believe this analysis may stray from our original intent and, indeed, be worthy of a separate follow-up paper addressing these important and well-received suggested research questions. Mention of this approach is added to the discussion section as a potential next step moving forward, as follows: "Furthermore, additional research, such as an examination of how participation in certain categories of collaboration within 1RBW are related to participation (or lack thereof) in other categories of collaboration, may help better elucidate the overall dynamics of this large collaborative." (Page 21, lines 467-470).

- If the authors decide to add some of these points, the Methods section would need updating, accordingly

Response: We have updated the methods section to include the following: "Measures of degree centrality (the immediate number of connections a given organization has to others) and betweenness centrality (a measure of positional importance of an organization within the network as being along pathway among other, otherwise potentially unconnected, organizations) were calculated to quantify organizational connectedness and positions within the various categories of collaboration[24]." (Page 11, lines 221-225).

- Tables 1 and 3 could be united in a single Table, leaving room for some of the analyses above. The same holds true for Tables 2 and 4.

Response: The tables were condensed as suggested.

Discussion

- The discussion seems comprehensive and covers a lot of topics – It definitely shows that the authors are well aware of the challenges in this field

Response: Thank you for this positive comment.

- What's missing (in the discussion and also potentially in the Introduction) is a clear statement on how representative the 1RBW network is of the global eye health landscape, which would tell the reader how relevant these findings are for global health policies

Response: We have added the following to the discussion: "We focused our analysis on the 1RBW network, which is documented in a publically available database (www.1rbw.org). It describes the global retinoblastoma burden and resources available to treat retinoblastoma, as reported by representatives of the treatment centers themselves. The 1RBW network is inclusive of the majority of global ophthalmic treatment centers, with countries lacking representation in the network expected to manage just 7% of the world's estimated retinoblastoma population (unpublished results)." (Pages 16-17, lines 348-351).

- A mention to (and a contrast with) the goals and findings of initiatives such as the Lancet Global Health Commission on Global Eye Health seems necessary (e.g. https://doi.org/10.1016/S2214-109X(20)30488-5), yet missing

Response: We have referenced the Lancet Commission on Global Eye Health as follows: "Consistent with the latter approach, the recent Lancet Commission on Global Eye Health posits that improving eye health is key to achieving the Sustainable Development Goals, framing it as a key global health issue[21]. The Commission emphasizes the importance of improving not just capacity, accessibility and quality of eye health services, but also the vital need for reliable data and innovation to drive progress[21]…Arguably, the majority of collaborations reported in this study could benefit from aligning their activities with a global health approach to more sustainably achieve their goals. (Pages 17-18, lines 365-375)

- Perhaps worth expanding/aligning to new potential results, if some of the additional points are addressed based on the comments above

Response: To the discussion, we have added the following: "Further, centrality measures from the most well-connected centers showing greater unidirectional out-degree interaction with much less in-degree interaction was suggestive of a power imbalance; this was not observed with a well-connected center in East Asia, which demonstrated bidirectional interactions of equivalent centrality. This exposes a striking irony: while the primary motivation behind collaboration was to promote patient survival, the apparent dominance of actors in HICs might inadvertently promote the very structural inequities that contribute to the poor outcomes observed for retinoblastoma in LMICs." (Page 18, lines 382-389).

- I found that two references (among others which the authors may know about) are missing and could add value to the paper if discussed in relation to the study results
1. https://doi.org/10.1186/1478-4491-12-44
2. https://doi.org/10.1093/heapol/czs031

Response: Thank you for suggesting these references. We discuss reference 1 and 2, respectively, and related publications, as follows: "Twinning/capacity building and joint planning activities reported in this study are most aligned with this model, as well as calls to improve human resources for eye health in LMICs[27], but represented the least common activities in 1RBW." (Page 17, lines 370-372) and "Social network analyses in health services research can uncover this type of information[15], and have proven insightful in the study of activities related to ophthalmology[16,17] and cancer[18,19] care." (Page 6, lines 103-105).

Reviewer 2
Global burden of retinoblastoma is in LMICs, where there is also a high mortality rate despite it being a curable condition. International collaborations through 1RBW aim to support optimal care and improve diagnosis and outcomes for retinoblastoma. This paper aims to explore the structure of global collaborations for retinoblastoma and how this can contribute to improved outcomes in order to identify where collaboration can be strengthened and replicated. This is a novel approach to exploring how collaboration can be strengthened for health outcomes, and could be applied to networks beyond retinoblastoma.

Introduction
Reference 4 weblink no longer correct

Response: Thank you for noting this. We have corrected the weblink in Reference 4.

It would be helpful if lines 90-91 could be elaborated slightly to provide some examples of how global collaboration can improve retinoblastoma outcomes (or of how such collaboration has improved outcomes in other conditions). Clearly this is one of the aims of the paper, but ten references have been given for collaborations that have shown progress towards improving outcomes – a brief summary here would be useful.

Response: We have expanded this section as follows: "Several international collaborations have shown progress towards improving retinoblastoma outcomes; activities range from implementing an awareness campaign to achieve early diagnosis[5], establishing shared care models[6], standard clinical protocols[7-9] or twinning programs[10-12] to improve health service delivery capacity, using telemedicine to access centralized expertise[13], and development of a multidisciplinary national retinoblastoma strategy which includes a combination of approaches[14]." (Page 6, lines 93-98).

Methods
It would be useful to have some further details on the sample. E.g., are the 1RBW centres all of similar size, are they urban/rural? And for the results, how do those who responded to the survey compare with those who did not? As it's quite a small sample size this information would be helpful in thinking about whether the results are likely to be similar for other centres.

Response: We have addressed this in the results section as follows: "All egos were secondary or tertiary facilities and only 2 were located in rural areas; this is consistent with the overall representation in 1RBW, as the majority of facilities able to treat retinoblastoma are located in urban areas, and primary facilities are able to do little more than offer a retinoblastoma diagnosis." (Page 12, lines 243-246).

There are a lot more respondents in China than elsewhere; is this related to the number of centres there or was there a particular effort to recruit there?

Response: This is based on the model of shared care that was happening in China at the time; one ophthalmologist from the main referral centre would travel to all peripheral centres to provide retinoblastoma care (rather than have patients travel to one tertiary centre).

Some treatment centres are not in 1RBW; how do centres get involved in that, does 1RBW represent the main centres in each country?

Response: We now include a description of 1RBW in the Methods section, "The analysis was focused on the 1RBW network (www.1rbw.org), which describes the global retinoblastoma burden and resources available at treatment centers around the world. The centers listed on www.1rbw.org were initially identified by searching relevant literature (e.g. publications, grey literature) and membership lists of professional organizations related to retinoblastoma, ocular oncology, or pediatric oncology. Physician representatives of each identified center were invited to self-report their data; participation was voluntary. Additionally, centers not identified through this approach were able to make a request to be included in the database. Though 1RBW does not represent an exhaustive list of all centers that manage retinoblastoma patients, it is estimated to represent the vast majority. At the time of the study, there were 170 centers listed on www.1rbw.org." (Page 8, lines 131-141).

Were respondents given a time frame for any collaborating activity (or just asked about start dates on ongoing activities)? Did you have any information on the person completing the survey (and did this make a difference to responses)?

Response: The respondents were not given a time frame for any collaborating activity, only asked about start dates. We did not collect additional information on the person or teams completing the survey.

Semi-structured interview sample – does 'extent of connectivity' mean that participants were recruited based on having few to many connections, or did this take into account their organisations' centrality? A bit more detail on sampling would be helpful here as well – how were the 23 chosen?

Response: We have addressed this above in response to a similar comment from Reviewer #1.

The topic guide focuses on asking about multiple partnerships (strongest partners/weakest partnerships), but little on the barriers to achieving partnership in the first place. Many of the egos only have one alter, so some of these questions seem like they'd be difficult to answer. Were participants only recruited if they had multiple partnerships?

Response: As indicated in a prior response, key informants represented those with many as well as few partnerships. We have added more detail on the semi-structured interview process as follows, "Owing to the semi-structured nature of the interview, questions were modified to match the qualities of the ego and partnership history they described. For example, a key informant with only one connection would not be asked to describe their strongest and weakest partnerships (Q2a and Q3a in Supplementary File 3), but instead asked to describe that connection as either strong or weak; subsequent questions would be modified accordingly." (Pages 10-11, lines 196-208).

It should be included in the methods that all interviews conducted in English. Perhaps more should be said about this in the limitations, particularly given the message of the paper being about the need for 'active efforts to include actors located at network peripheries' and that 'members of the retinoblastoma community remain aware of power imbalances' – is there some irony in this message given that those at the periphery may have been less able to be involved in the study? Do researchers see themselves as part of this network, and if so by only conducting the research in English does this go against the very messages coming out of the study?

Response: Thank you for pointing this out. We have now revised to:

- indicate that the interviews were conducted in English (Page 11, line 209);
- add a section in the methods to outline the positionality of authors (Page 9, lines 161-171); and
- expand the limitations section as follows: "The rather uniform positionality of the authors is also a limitation of this study; inclusion of more diverse study team members (e.g. representatives of LMICs or secondary retinoblastoma centers) or conducting the study in additional languages may have yielded richer insights." (Page 21, lines 450-453)

Reciprocity of relationships was assumed, but surely this could have been checked since many of the reported partnerships were others in the sample?

Response: A challenge we have encountered in other similar studies is the possibility that the individual or individuals representing an organization participating in survey may not fully appreciate the full range of partnerships or collaborations their organization participates in. In these previous and ongoing studies, we have opted to take on the assumption that reciprocity exists, as these different types of collaboration measured in the network survey are not trivial (e.g, sharing resources and information, referring patients, etc.), and likely if mentioned by one party, the relationship does, in fact, exist.

Results
Although 62% of 1RBW are represented in the study, some of these did not actually provide information on their collaborations (& reciprocity was assumed). Though I note this is mentioned in the limitations section.

Response: Indeed, this is noted as a limitation of the study.

Figure 1 – it's difficult to differentiate between node size; could you make the bigger slightly bigger?
It's not clear from Figure 1 which egos had responded to the survey and which were only identified by others.

Response: We thank the reviewer for their comment, and we have revised figure 1 so that it can be more readable, per the reviewer's suggestion.

That there was no snowball sampling from responses should be highlighted as a limitation in the paper (it does not attempt to reflect a whole network), and a note should be added to Figure 1 to highlight that some

centres/organisations have fewer collaborations than likely in reality because they did not provide information about their collaborations.

Response: We have expanded the limitations section as follows, "A limitation of this study was that just 56 of the 170 centers (33%) listed on *1RBW* participated in the study, and we did not perform snowball sampling of identified alters outside of *1RBW*, meaning that our analysis may have missed relevant collaborative activities." (Page 20, lines 445-447).

Thematic analysis provides a fairly descriptive overview of the themes rather than really going into any depth about participants' views on the issues that are raised in the discussion. Given that the discussion goes into some detail on issues of power in the network, it would have been interesting to have presented some more in-depth findings related to this (from different veiwpoints) in the results. As it is, the results section provides a summary of interviews rather than any analysis backed up with evidence (e.g., quotes, though I note these are in the supplementary file, if word count allowed it would have been good to see some of these making up the main points in the findings section).

Response: The quotes are provided in a supplementary file due to word count limitations in the manuscript.

Discussion
The authors state that connectivity of nodes is an approximate measure of their power and strength within networks, yet this does not appear to be something that was asked about in the interviews (or at least not reported in much detail). This is unfortunate as the interviews were conducted after the survey and would have been expected to probe into the relationships uncovered in the network analysis.

Response: We agree that this analysis would have added strength to our manuscript. We hope that the addition of the centrality measures sheds light into this issue (see response to previous reviewer, above).

This is an interesting discussion around the issues touched upon in the findings, but could be expanded to provide more detail on how this research will be taken forward; how will the research contribute to "strive towards shifting the paradigm on which collaborations are currently modelled". The dissemination of the work could support the recommendations (for organisations to be aware of power imbalances within the existing network, the need for effort to re-design partnership activities to avoid perpetuating structural and social inequities). E.g., What could you do with the network analysis? Are there any network strengthening interventions, or dissemination activities planned with 1RBW to determine how to use these findings to support collaboration? It's not clear if the collaborative study that has started since this network analysis drew upon these findings at all (line 308). From the survey it is clear that there are organisations not in 1RBW but are nonetheless important members of the community. Does the network need to be expanded in some way?

Response: Thank you for these insights. We have made the following revisions:

- with respect to the collaborative study (reference #39), we have clarified, "However, an examination of the investigators who declined to collaborate in this study, and their reasons why, could reveal important structural inequities that still need to be addressed in order to facilitate an inclusive environment for research participation and leadership." (page 20, lines 433-436)
- we have addressed dissemination of this work and network strengthening interventions as follows: "In summary, the findings suggest that there exists a large group of actors working to reduce retinoblastoma mortality, but there is room to expand connectivity and interaction through active efforts to include actors located at network peripheries. This can be achieved by dissemination of this and other similar work focused on achieving representation and equity in international partnerships[41], which could stimulate important discussion, knowledge exchange and action in global retinoblastoma activities. Professional bodies in pediatric and ocular oncology could play a role in such efforts, for example by inviting and supporting underrepresented actors to attend international conferences to increase their visibility and share their work. It has become increasingly common for retinoblastoma conference sessions to highlight global retinoblastoma partnerships, however, they still remain largely led by HIC

speakers focused on clinical impact of activities, with little attention paid to how partnerships are developed, structured and maintained. Professional bodies can support the latter by making it a requirement to include a statement of reflexivity with respect to international partnerships, as has been suggested for research publications involving the same[42]. Furthermore, additional research, such as an examination of how participation in certain categories of collaboration within 1RBW are related to participation (or lack thereof) in other categories of collaboration, may help better elucidate the overall dynamics of this large collaborative. Going forward, we recommend that members of the global retinoblastoma community are made aware of power imbalances inherent within the existing network, and make concerted effort to research and re-design partnership activities to avoid perpetuating structural and social inequities." (Pages 20-21, lines 454-475)

In addition to the revisions outlined above, we have also included a data availability statement on the title page.

Thank you once again for considering our manuscript for publication in PLOS Global Public Health.

Sincerely (on behalf of all co-authors),

Helen Dimaras, PhD
Associate Professor,
Department of Ophthalmology & Vision Sciences, Faculty of Medicine, &
Division of Clinical Public Health, Dalla Lana School of Public Health, University of Toronto
Scientist
Child Health Evaluative Sciences &
Center for Global Child Health, SickKids Research Institute
Director of Global Eye Health Research,
Department of Ophthalmology & Vision Sciences, The Hospital for Sick Children
555 University Ave, Toronto, Ontario M5G 1X8 T: 416-813-7654 x201876; E: helen.dimaras@sickkids.ca
