# Supplementary material for: Characterization of international partnerships in global retinoblastoma care and research: A network analysis
Source: PLOS Glob Public Health. 2021 Dec 16;1(12):e0000125. doi: 10.1371/journal.pgph.0000125 (PMC10021644; doi:10.1371/journal.pgph.0000125)
Supplement: Attachment — Submitted filename: Dimaras Response to Reviewers2 PLOS GPH 15NOV2021 v1.pdf [file pgph.0000125.s010.pdf]

Ophthalmology & Vision Sciences
UNIVERSITY OF TORONTO

Catherine Kyobutungi, MD, PhD
Editor-in-Chief, PLOS Global Public Health
African Population and Health Research Center
Nairobi, Kenya

15 November 2021

Dear Dr. Catherine Kyobutungi,

RE: "Characterization of international partnerships in global retinoblastoma care and research: a network analysis" by Girdler et al.

Thank you for the opportunity to submit a revision of our manuscript entitled "Characterization of international partnerships in global retinoblastoma care and research: a network analysis". We respond to the reviewer comments in a point-by-point fashion below.

Reviewer #1:
The authors made an excellent work in addressing comments to the previous version of the manuscript. The paper is vastly improved and will make a great contribution to the literature. I only have two last minor comments, related to the centrality measures introduced. Firstly, can the authors report the characteristics of the most central nodes (centrality measure value, geographical region, type of organisation, etc) in terms of degree and betweenness centrality?

Response: We thank the reviewer for their gracious comments, as well as their earlier comments that helped to further improve our work. For the reviewers first minor concern, we have added more details about the three most central nodes to the results section, including centrality measures, more detail about their geographic region, and type of organization. (Page 14, line 251-263).

Secondly, do centrality rankings differ across centrality measures? What does that imply? Since you computed it, why don't you explicitly discuss betweenness centrality? I believe addressing these points would allow an extra step up in terms of interpreting the eyecare partnerships represented, and at the same time make an even better use of the rich network data collected.

Response: We thank the reviewer for this comment. Indeed, there are similarities between degree and betweenness centrality, which has important implications to the connectedness of centers within the network. Not only are these centers highly connected, but they are more often in the direct path between other centers who are not directly connected. Implications of this feature of the network are that these three central organizations not only connect with most member centers, but facilitate the existence of connections among other organizations with each other. We have made additions to the results (Page 14, lines 251-263) and discussion sections (Page 19, 374-378) reflecting this.

Reviewer #2: *Thank you for responding to comments and making edits to the paper, which have been very helpful to my understanding of the work. I think most have been addressed but there are a few outstanding points/questions:*
1. Regarding the response to the question that I previously asked about the number of respondents in China (from supplementary file S3), and that participants were eligible if they were a healthcare professional (e.g. oncologist, ophthalmologist) leading a retinoblastoma team at a treatment centre, does this mean that one ophthalmologist responded to the survey 18 times? If so, I think this is important to note, given the implications (particularly that there is a link between each of these centres as there is one ophthalmologist travelling between them).

Response: We wish to clarify that while one ophthalmologist from the main referral site traveled to other secondary treatment centers, they were not the only clinician providing care at those sites. Each treatment centre had an independent www.1rbw.org account and access to this research study. While it is possible that the ophthalmologist who provided care at multiple sites was consulted in the process to populate the survey (as we instructed each participating centre to do) it is unlikely that they filled out the survey

responses for multiple centers themselves. We have clarified the unique context of the Chinese collaboration in the manuscript (Page 21, lines 430-434).

2. Regarding the point about the time frame for collaborating activity – the authors' response indicates that the activity could be historical and perhaps not reflect an ongoing collaboration between centres. This may be considered a limitation of the survey, and should be noted in the paper.

Response: We have added the limitation as follows: "In addition, since participants were asked to report on activities in the last 12 months but were not asked to provide an end date (if relevant), it is possible that some activities represented one-time activities rather than ongoing collaborations." (Pages 21-22, lines 440-443).

3. The comments about the results section providing a descriptive overview of themes, and about the lack of information from the interviews about power issues (which was identified and highlighted in the discussion) remain. The additional information on centrality helps, but it is a limitation that the qualitative interviews and analysis did not delve into this issue, particularly given the focus given to it in the discussion.

Response: Thank you for this comment. The interviews did not provide explicit views on power balances or imbalances in the identified partnerships. Rather, as we have noted in the results section, interviews commented on the presence or absence of MoUs, the differences in partners in terms of who holds the financial resources and perceived expertise, and differing views on benefits of partnerships. Some of these accounts are suggestive of potential power imbalances within partnerhips, which we note in the discussion, as a potential interpretation of our findings. We have revised the results and discussion to more clearly articulate how the results support the interpretation in the discussion (Pages 15-16, lines 298-331), and added a few more representative quotes to Supplementary File S7.

Thank you once again for considering our manuscript for publication in PLOS Global Public Health.

Sincerely (on behalf of all co-authors),

Helen Dimaras, PhD
Associate Professor,
Department of Ophthalmology & Vision Sciences, Faculty of Medicine, &
Division of Clinical Public Health, Dalla Lana School of Public Health, University of Toronto
Scientist
Child Health Evaluative Sciences &
Center for Global Child Health, SickKids Research Institute
Director of Global Eye Health Research,
Department of Ophthalmology & Vision Sciences, The Hospital for Sick Children
555 University Ave, Toronto, Ontario M5G 1X8 T: 416-813-7654 x201876; E: helen.dimaras@sickkids.ca
